# Differentially expressed proteins underlying childhood cortical dysplasia with epilepsy identified by iTRAQ proteomic profiling

**DOI:** 10.1371/journal.pone.0172214

**Published:** 2017-02-21

**Authors:** Lu Qin, Xi Liu, Shiyong Liu, Yi Liu, Yixuan Yang, Hui Yang, Yangmei Chen, Lifen Chen

**Affiliations:** 1 Department of Neurology, The Second Affiliated Hospital of Chongqing Medical University, Chongqing, People’s Republic of China; 2 Department of Neurosurgery, The Xinqiao Hospital of Third Military Medical University, Chongqing, People’s Republic of China; 3 Department of Infectious Disease, The Second Affiliated Hospital of Chongqing Medical University, Chongqing, People’s Republic of China; Boston Children's Hospital / Harvard Medical School, UNITED STATES

## Abstract

Cortical dysplasia accounts for at least 14% of epilepsy cases, and is mostly seen in children. However, the understanding of molecular mechanisms and pathogenesis underlying cortical dysplasia is limited. The aim of this cross-sectional study is to identify potential key molecules in the mechanisms of cortical dysplasia by screening the proteins expressed in brain tissues of childhood cortical dysplasia patients with epilepsy using isobaric tags for relative and absolute quantitation-based tandem mass spectrometry compared to controls, and several differentially expressed proteins that are not reported to be associated with cortical dysplasia previously were selected for validation using real-time polymerase chain reaction, immunoblotting and immunohistochemistry. 153 out of 3340 proteins were identified differentially expressed between childhood cortical dysplasia patients and controls. And FSCN1, CRMP1, NDRG1, DPYSL5, MAP4, and FABP3 were selected for validation and identified to be increased in childhood cortical dysplasia patients, while PRDX6 and PSAP were identified decreased. This is the first report on differentially expressed proteins in childhood cortical dysplasia. We identified differential expression of FSCN1, CRMP1, NDRG1, DPYSL5, MAP4, FABP3, PRDX6 and PSAP in childhood cortical dysplasia patients, these proteins are involved in various processes and have various function. These results may provide new directions or targets for the research of childhood cortical dysplasia, and may be helpful in revealing molecular mechanisms and pathogenesis and/or pathophysiology of childhood cortical dysplasia if further investigated.

## Introduction

Cortical dysplasia is a common cause of epilepsy and accounts for at least 14% of epilepsy cases [1], among whom more than 40% were refractory epilepsy [2]. It happens mostly in childhood [3]. Although previous researches revealed several genetic and acquired causes of childhood cortical dysplasia (CCD) and the mechanisms of its epileptogenesis [4], our understanding of molecular mechanisms and pathogenesis underlying CCD with epilepsy is still limited.

In previous studies, only a few analyzed the proteomics of epilepsy patients using brain tissues or cerebrospinal fluid [5, 6, 7]. However, the proteomics or transcriptomics of CCD with epilepsy has not been analyzed, especially in brain tissues of CCD patients. Isobaric tags for relative and absolute quantitation (iTRAQ) is a comparative proteomic approach that can analyze up to 8 samples in one experiment, and is widely used in proteomic researches in different diseases [8, 9]. Moreover, This study screened the differentially expressed proteins in brain tissues of CCD patients with epilepsy compared to traumatic intracranial hypertension (TIH) patients using iTRAQ-based tandem mass spectrometry and selected several proteins that are differentially expressed or unreported associated with CCD previously for validation using real-time quantitative polymerase chain reaction (qPCR) analysis, immunoblotting and immunohistochemistry. Our result suggests that 153 out of 3340 proteins were diffrentially expressed in patients with CCD compared to controls, and these proteins are mainly involved in mechanisms of catalytic activity, binding, molecule-structuring activity, transporter activity, and enzyme regulation activity. Among these 153 proteins, 8 proteins that have not been associated with CCD, but participate in CCD-related biological processes or have CCD-related molecular functions according to Gene Oncology, including NDRG1, FSCN1, FABP3, DPYSL5, PSAP, MAP4, CRMP1, and PRDX6 were selected and validated.

## Materials and methods

### Patients and tissue preparation

The study protocol was approved by the Ethics Committee of the Second Affiliated Hospital of Chongqing Medical University (2013–026), and the study was conducted according to the principle expressed in the Declaration of Helsinki. Written consents were obtained from patients and/or their legal guardians. No author but S. L. and H. Y. had access to information that could identify individual participants during data collection, while no author had access to such information after data collection.

All the included CCD patients were recruited from 2013 August to 2014 December, and were preoperatively assessed with detailed history, neurological examination, neuropsychological test, ictal and interictal electroencephalography and MRI together with intraoperative electrocorticography for diagnosis and localization of lesions. After surgery, brain tissues from CCD patients were diagnosed by neuropathologists according to consensus of International League Against Epilepsy [10]. All included TIH patients underwent surgery had no history of neurological diseases, and the resected brain tissues were histologically normal. The resected tissues were immediately immersed into liquid nitrogen and strored at -80°C. Brain tissues of sex- and age-matched 8 CCD patients and 8 controls were selected for iTRAQ, brain tissues from the rest 15 CCD patients and 15 controls were used for qPCR, immunoblotting and immunohistochemistry.

### Sample preparation and iTRAQ reagents labeling

Total proteins were extracted with iTRAQ lysis buffer. The concentration of proteins was measured using 2-D Quant Kit (Amersham Biosciences, Uppsala, Sweden). Equal amount of proteins from each group were mixed. The pooled samples were subjected to iTRAQ labeling according to the iTRAQ kit protocol (Applied Biosystems, Framingham, MA, USA). Briefly, 2 μl reducing reagent was added to 200 μg protein and centrifuged, then it was incubated at 37°C for 1 hour. 1 μl of Cysteine-Blocking Reagent was added for cysteine blocking. Each protein sample was digested into peptide with 4 μg Trypsin overnight at 37°C. iTRAQ reagents were dissolved in isopropanol, and then mixed with the corresponding sample followed by incubation at room temperature for 3 hours. Samples from CCD patients were labeled with 118 tag and 121 tag, and samples from controls were labeled with 117 tag and 119 tag. All the iTRAQ reagent-labeled samples were then combined. [11, 12]

### Peptide fractionation with Isoelectric Focusing (IEF)

The labeled peptides samples were fractionated by IEF on immobilized pH gradient as described previously [13–15]. Briefly, the labeled peptides were dissolved in urea and Pharmalyte solution, applied to IPG strips (pH 3–10), and then focused with an IPGphor system (GE Healthcare Life Sciences Amersham Biosciences, Pittsburg, PA, USA) at 68 kVh. The IPG strips were cut into 36 pieces (0.5cm per piece). Peptides in each pieces was extracted with 0.1% formic acid and 2% acetonitrile and lyophilized and desalted with a C18 Discovery DSC-18 SPE column (Sigma-Aldrich). The desalted peptides were lyophilized again and stored at -20°C for mass spectrometry analysis.

### Mass spectrometry and gene oncology analysis

Mass spectrometry was performed with liquid chromatography coupled inline to a QStar mass spectrometer (Applied Biosystems, Framingham. MA, USA). Desalted peptides were reconstituted in a solution containing 0.1% formic acid and 2% acetonitrile, half of which was delivered into a trap column by an online capillary liquid chromatography system (Dionex Ultimate 3000, Amsterdam, The Netherlands). The peptide mixture were automatically separated on a C18-PepMap column (ThermoFisher Dionnex, Sunnyvale, CA, USA) at 0.3 μl/min. The eluent was analysed by OStar Elite Hybrid ESI Quadrupole time-of-flight tandem mass spectrometer (Applied Biosystems, Framingham. MA, USA) in an information-dependent acquisition mode. Mass spectrometer data acquisition was performed in the positive ion mode, with a selected mass range of 300–1800 *m/z*. A setting of 2 s was used as the total time for MS/MS events. The two charged peptides which were most abundant, with more than 20 counts, were selected for MS/MS and dynamically excluded for 30 s with ± 50 mDa mass tolerance.

Peptide identification and quantification was performed by ProteinPilot software (Applied Biosystems, Framingham, MA, USA). The search was performed using the International Protein Index (IPI) human database v3.87. Cysteine modification by MMTS was specified as a fixed modification [16].

For protein identification, a generally accepted standard which has been widely used in identifying and quantifying proteins with iTRAQ was taken [17–20]. The protein threshold was set to achieve 95% confidence, False discovery rate (FDR) statistics and 1.3-fold change cut-offs were used to classify the protein expressions as up-regulated (FDR<0.05 and iTRAQ fold-changes above 1.3) or down-regulated (FDR<0.05 iTRAQ and fold-changes below 0.77). For technical variation, while an analysis of repeated iTRAQ experiments established the technical variability to be not more than 30%. The gene oncology of each differentially expressed proteins was searched and classified using PANTHER classification system (www.pantherdb.org). 8 differentially expressed proteins which have not been reported associated with CCD, but may participate in CCD-related biological processes or have CCD-related molecular functions according to previous literature were selected for further validation.

### Real-time qPCR analysis

Total RNA was extracted using Trizol (Thermofisher, Waltham, USA). Extracted RNA was reverse transcribed into cDNA by A3500 Reverse Transcription System (Promega, Madison, WI, USA). qPCR was performed using TaqMan GeneExpression Kit in ABI 7900HT system. The sequences of primers (OriGene Technologies, Inc. Rockville, USA) were NDRG1 (HP209104), FSCN1 (HP206673), FABP3 (HP207465), DPYSL5 (HP213501), CRMP1 (HP232913), PRDX6 (HP208150), PSAP (HP231407), MAP4 (HP206072), and β-Actin (HP204660). The mRNA expression level were analyzed using ΔΔCt method.

### Immunoblotting analysis

Total proteins were extracted with RIPA Lysis Buffer and the concentrations were determined with BCA Kit (Beyotime, Haimen, China). Protein samples were loaded to 10% SDS-PAGE gel for electrophoresis and transferred to PVDF membranes. The membranes were incubated in 0.4% gelatin for 1 hour at room temperature and then incubated in primary antibodies (CRMP1, DPYSL5, FSCN1, NDRG1, PRDX6) (1:1000–1:10000 dilution, Abcam, Cambridge, UK) at 4°C overnight. After washed with TBST buffer, the membranes were incubated in HRP-conjugated secondary antibody (1:5000 dilution) for 1h at room temperature. The protein bands were visualized using ECL detection system (Millipore, Germany) and analyzed using Quantity One software (Bio-Rad Laboratories, Hercules, CA, USA).

### Immunohistochemistry

Brain tissues from CCD patients and controls were fixed with 4% paraformaldehyde overnight at 4°C, then routinely embedded in paraffin and sectioned. After dewaxing and rehydration, the sections were boiled in citrate buffer (pH 6.0) in microwave oven for 20 min for antigen retrieval. Endogenous peroxidase activity was quenched by treatment with 3% H_2_O_2_ for 10 min. The sections were blocked with goat serum for 30 min and incubated at 4oC overnight with primary antibodies (CRMP1 1:250, DPYSL5 1:100 and FSCN1 1:250). Then sections were incubated in goat-anti-rabbit secondary antibodies (30 min, 37°C) and visualized using DAB (ZSGB-Bio, Beijing, China). After counterstain with hematoxylin and dehydrated, sections were evaluated under microscope. A semi-quantitative score was applied to the images obtained from the microscope inspection. The staining intensity ranged from 1 to 3 and the percentage of positive cells was measured manually in a range from 0 to 100%. Staining intensity (1–3) was multiplied by the percentage of positive cells (0–100) to obtain a final score ranging from 0 to 300 [21]. This method was repeated three times for the immunohistochemistry of each antibody in the brain tissue samples.

### Statistical analysis

Data was expressed as mean±SD, FDR statistics was used to identified differentially expressed proteins, FDR<0.05 was considered statistically significant. Intergroup differences in immunoblotting and immunohistochemistry between the CCD group and the control group were analyzed using t test or rank sum test. p<0.05 was considered statistically significant.

## Results

### Demographics and clinical characters of patients

23 CCD patients (11 female, age 6.96±3.70, disease course 2.76±1.61 years) and 23 TIH patients (8 Female, age 7.22±3.10 years) who underwent surgery were included in this study. (Table 1)

**Table 1:** Patient demographics and clinical characteristics.

Characteristic	CCD group	Controls
Age (year), mean ± SD	6.96±3.70	7.22±3.10
Sex (male/female)	12/11	15/8
Diagnosis	CCD with epilepsy	Trauma
Tissue pathology	Cortical dysplasia	Normal
Resection tissue	Neocortex	Neocortex

CCD, Childhood cortical dysplasia.

### Differentially expressed proteins revealed by iTRAQ analysis

Brain tissues of 16 randomly selected patients (n = 8 for each group) were analyzed with iTRAQ. In total, 3440 proteins were found with 95% confidence, among which 153 proteins were differentially expressed (FDR<0.05), including 64 up-regulated and 89 down-regulated proteins (Tables 2 & 3).

**Table 2:** 64 up-regulated proteins in childhood cortical dysplasia patients with epilepsy compared to controls by iTRAQ.

Accession	Gene Name	Protein	118:117	121:119
IPI:IPI00478003.3	A2M	Alpha-2-macroglobulin	6.607	6.546
IPI:IPI00335509.3	DPYSL5	Dihydropyrimidinase-related protein 5	5.152	4.966
IPI:IPI00215801.1	RBM39	Isoform 2 of RNA-binding protein 39	4.966	4.246
IPI:IPI00291932.1	ACAN	Isoform 3 of Aggrecan core protein	3.311	3.698
IPI:IPI00923597.2	NDRG1	cDNA FLJ39243 fis, clone OCBBF2008283, highly similar to Protein NDRG1	3.631	3.597
IPI:IPI00647915.1	TAGLN2	TAGLN2 24 kDa protein	2.965	3.597
IPI:IPI00218993.1	HSPH1	Isoform Beta of Heat shock protein 105 kDa	3.436	3.532
IPI:IPI00220213.2	TNC	Isoform 4 of Tenascin	3.342	3.404
IPI:IPI00744780.2	BCAS1	Isoform 2 of Breast carcinoma-amplified sequence 1	3.767	3.311
IPI:IPI00026237.1	MAG	Myelin-associated glycoprotein	3.467	3.251
IPI:IPI00640953.1	SIRT2	Sirtuin-2	3.221	2.992
IPI:IPI00219684.3	FABP3	Fatty acid-binding protein, heart	2.992	2.831
IPI:IPI00641181.5	MARCKSL1	MARCKS-related protein	2.704	2.831
IPI:IPI00415014.3	MAP1LC3A	Isoform 1 of Microtubule-associated proteins 1A/1B light chain 3A	3.221	2.729
IPI:IPI00553211.1	ERMN	Isoform 2 of Ermin	2.729	2.704
IPI:IPI00032958.3	ANLN	Isoform 2 of Actin-binding protein anillin	2.754	2.582
IPI:IPI00298497.3	FGB	Fibrinogen beta chain	2.630	2.559
IPI:IPI00295777.6	GPD1	Glycerol-3-phosphate dehydrogenase [NAD+], cytoplasmic	2.805	2.489
IPI:IPI00556376.2	CRMP1	dihydropyrimidinase-related protein 1 isoform 1	2.630	2.489
IPI:IPI00295469.5	CPNE6	cDNA FLJ55997, highly similar to Copine-6	2.399	2.270
IPI:IPI00854567.3	KIAA1598	Isoform 2 of Shootin-1	2.312	2.249
IPI:IPI00022463.1	TF	Serotransferrin	2.270	2.249
IPI:IPI00173346.3	PGM2L1	Glucose 1,6-bisphosphate synthase	1.803	2.188
IPI:IPI00059135.1	PPP1R14A	Isoform 1 of Protein phosphatase 1 regulatory subunit 14A	2.606	2.148
IPI:IPI00157414.3	ENPP6	Ectonucleotide pyrophosphatase/phosphodiesterase family member 6	2.312	2.109
IPI:IPI00396130.5	SRCIN1	Isoform 4 of SRC kinase signaling inhibitor 1	2.128	2.089
IPI:IPI00856045.1	AHNAK2	Isoform 1 of Protein AHNAK2	2.089	2.089
IPI:IPI00329719.1	MYO1D	Myosin-Id	2.291	2.070
IPI:IPI00007702.1	HSPA2	Heat shock-related 70 kDa protein 2	2.070	2.070
IPI:IPI00027223.2	IDH1	Isocitrate dehydrogenase [NADP] cytoplasmic	2.070	2.051
IPI:IPI00747810.2	FSCN1	FSCN1 protein (Fragment)	2.270	1.977
IPI:IPI00940816.2	ARHGEF2	Isoform 3 of Rho guanine nucleotide exchange factor 2	2.070	1.977
IPI:IPI00021841.1	APOA1	Apolipoprotein A-I	2.089	1.905
IPI:IPI00878314.1	MAP4	110 kDa protein	2.070	1.905
IPI:IPI00553177.1	SERPINA1	Isoform 1 of Alpha-1-antitrypsin	2.148	1.888
IPI:IPI00029111.3	DPYSL3	Collapsin response mediator protein 4 long variant	1.941	1.871
IPI:IPI00873622.3	WDR1	Putative uncharacterized protein WDR1	1.786	1.837
IPI:IPI00045051.3	PURB	Transcriptional activator protein Pur-beta	1.941	1.803
IPI:IPI00760925.2	MYO18A	Isoform 3 of Myosin-XVIIIa	1.820	1.786
IPI:IPI00554737.3	PPP2R1A	Serine/threonine-protein phosphatase 2A 65 kDa regulatory subunit A alpha isoform	1.500	1.770
IPI:IPI00004560.1	DCLK1	Isoform 2 of Serine/threonine-protein kinase DCLK1	1.854	1.706
IPI:IPI00022388.2	DPYSL4	Dihydropyrimidinase-related protein 4	1.837	1.706
IPI:IPI00304409.3	CARHSP1	Calcium-regulated heat stable protein 1	1.706	1.690
IPI:IPI00926256.1	SLC4A1	Band 3 anion transport protein	1.629	1.690
IPI:IPI00010133.3	CORO1A	Coronin-1A	1.820	1.660
IPI:IPI00021766.5	RTN4	Isoform 1 of Reticulon-4	1.871	1.629
IPI:IPI00218414.5	CA2	Carbonic anhydrase 2	1.871	1.629
IPI:IPI00916847.1	OLA1	OLA1 47 kDa protein	2.109	1.614
IPI:IPI00186966.3	BIN1	Isoform IIA of Myc box-dependent-interacting protein 1	1.803	1.585
IPI:IPI00965262.1	CLASP2	166 kDa protein	1.754	1.585
IPI:IPI00185159.7	BAIAP2	Isoform 4 of Brain-specific angiogenesis inhibitor 1-associated protein 2	1.614	1.528
IPI:IPI00940222.1	AKAP12	Isoform 3 of A-kinase anchor protein 12	1.600	1.500
IPI:IPI00479514.2	CACNA2D1	Isoform 2 of Voltage-dependent calcium channel subunit alpha-2/delta-1	1.675	1.486
IPI:IPI00017895.3	GPD2	Isoform 1 of Glycerol-3-phosphate dehydrogenase, mitochondrial	1.600	1.486
IPI:IPI00385612.2	SLC8A2	Putative uncharacterized protein DKFZp761D171	1.514	1.486
IPI:IPI00294187.1	PADI2	Protein-arginine deiminase type-2	1.459	1.486
IPI:IPI00455620.3	RUFY3	protein RUFY3 isoform 1	1.445	1.486
IPI:IPI00942902.1	GDA	Guanine deaminase	1.472	1.472
IPI:IPI00022774.3	VCP	Transitional endoplasmic reticulum ATPase	2.965	1.459
IPI:IPI00910602.1	NEFH	Isoform 1 of Neurofilament heavy polypeptide	1.614	1.445
IPI:IPI00465436.4	CAT	Catalase	1.644	1.380
IPI:IPI00159927.2	NCAN	Neurocan core protein	1.675	1.330
IPI:IPI00306667.5	CNP	Isoform CNPII of 2',3'-cyclic-nucleotide 3'-phosphodiesterase	1.393	1.318
IPI:IPI00456623.2	BCAN	Isoform 1 of Brevican core protein	1.432	1.306

**Table 3:** 89 down-regulated proteins in childhood cortical dysplasia patients with epilepsy compared to controls by iTRAQ.

Accession	Gene Name	Protein	118:117	121:119
IPI:IPI00480085.6	DNM3	Putative uncharacterized protein DNM3	0.711	0.738
IPI:IPI00909720.1	PSD3	cDNA FLJ54694, highly similar to Pleckstrin and Sec7 domain-containing protein3	0.679	0.738
IPI:IPI00219446.5	PEBP1	Phosphatidylethanolamine-binding protein 1	0.679	0.731
IPI:IPI00418471.6	VIM	Vimentin	0.738	0.718
IPI:IPI00789794.1	DLG4	disks large homolog 4 isoform 2	0.731	0.711
IPI:IPI00024990.6	ALDH6A1	Methylmalonate-semialdehyde dehydrogenase [acylating], mitochondrial	0.745	0.705
IPI:IPI00011515.1	PACSIN1	Protein kinase C and casein kinase substrate in neurons protein 1	0.745	0.698
IPI:IPI00926312.1	OGDH	oxoglutarate dehydrogenase isoform 3 precursor	0.745	0.685
IPI:IPI00031804.1	VDAC3	Isoform 1 of Voltage-dependent anion-selective channel protein 3	0.698	0.685
IPI:IPI00007682.2	ATP6V1A	V-type proton ATPase catalytic subunit A	0.766	0.673
IPI:IPI00003968.1	NDUFA9	NADH dehydrogenase [ubiquinone] 1 alpha subcomplex subunit 9, mitochondrial	0.649	0.673
IPI:IPI00217871.4	ALDH4A1	Delta-1-pyrroline-5-carboxylate dehydrogenase, mitochondrial	0.759	0.667
IPI:IPI00954954.1	CLU	clusterin isoform 3	0.752	0.667
IPI:IPI00299402.1	PC	Pyruvate carboxylase, mitochondrial	0.679	0.667
IPI:IPI00007812.1	ATP6V1B2	V-type proton ATPase subunit B, brain isoform	0.711	0.661
IPI:IPI00026216.4	NPEPPS	Puromycin-sensitive aminopeptidase	0.673	0.655
IPI:IPI00386271.4	SLC25A12	Calcium-binding mitochondrial carrier protein Aralar1	0.698	0.649
IPI:IPI00167215.6	HEPACAM	Isoform 1 of Hepatocyte cell adhesion molecule	0.619	0.637
IPI:IPI00940744.1	NDUFS1	NADH-ubiquinone oxidoreductase 75 kDa subunit, mitochondrial	0.608	0.637
IPI:IPI00009439.1	SYT1	Synaptotagmin-1	0.738	0.625
IPI:IPI00300568.4	SYN1	Isoform IA of Synapsin-1	0.614	0.625
IPI:IPI00017855.1	ACO2	Aconitate hydratase, mitochondrial	0.711	0.608
IPI:IPI00219078.5	ATP2A2	Isoform 1 of Sarcoplasmic/endoplasmic reticulum calcium ATPase 2	0.631	0.592
IPI:IPI00847322.1	SOD2	superoxide dismutase 2, mitochondrial isoform A precursor	0.766	0.586
IPI:IPI00018342.5	AK1	Adenylate kinase isoenzyme 1	0.679	0.575
IPI:IPI00009532.5	ABAT	cDNA FLJ56034, highly similar to 4-aminobutyrate aminotransferase, mitochondrial	0.597	0.570
IPI:IPI00218660.3	ITPR1	Isoform 4 of Inositol 1,4,5-trisphosphate receptor type 1	0.470	0.570
IPI:IPI00873201.1	PSAP	Isoform Sap-mu-6 of Proactivator polypeptide	0.457	0.565
IPI:IPI00328156.9	MAOB	Amine oxidase [flavin-containing] B	0.619	0.555
IPI:IPI00219219.3	LGALS1	Galectin-1	0.586	0.550
IPI:IPI00746777.3	ADH5	Alcohol dehydrogenase class-3	0.515	0.550
IPI:IPI00016801.1	GLUD1	Glutamate dehydrogenase 1, mitochondrial	0.679	0.545
IPI:IPI00941244.1	AQP4	33 kDa protein	0.673	0.545
IPI:IPI00006663.1	ALDH2	Aldehyde dehydrogenase, mitochondrial	0.643	0.545
IPI:IPI00028520.2	NDUFV1	Isoform 1 of NADH dehydrogenase [ubiquinone] flavoprotein 1, mitochondrial	0.565	0.545
IPI:IPI00946334.1	NDUFS2	dehydrogenase (ubiquinone) Fe-S protein 2 isoform 2 precursor	0.530	0.545
IPI:IPI00643720.3	OGDHL	2-oxoglutarate dehydrogenase-like, mitochondrial	0.614	0.540
IPI:IPI00023591.1	PURA	Transcriptional activator protein Pur-alpha	0.718	0.535
IPI:IPI00009771.6	LMNB2	Lamin-B2	0.625	0.530
IPI:IPI00015602.1	TOMM70A	Mitochondrial import receptor subunit TOM70	0.597	0.530
IPI:IPI00011229.1	CTSD	Cathepsin D	0.575	0.530
IPI:IPI00013508.5	ACTN1	Alpha-actinin-1	0.530	0.530
IPI:IPI00021088.1	KCNAB2	Isoform 1 of Voltage-gated potassium channel subunit beta-2	0.373	0.530
IPI:IPI00383807.1	SLC4A4	Electrogenic Na+ bicarbonate cotransporter (Fragment)	0.631	0.525
IPI:IPI00479877.4	ALDH9A1	4-trimethylaminobutyraldehyde dehydrogenase	0.488	0.520
IPI:IPI00413060.1	SYNPO	Isoform 3 of Synaptopodin	0.461	0.520
IPI:IPI00004358.4	PYGB	Glycogen phosphorylase, brain form	0.685	0.515
IPI:IPI00007087.4	FBXO2	F-box only protein 2	0.501	0.511
IPI:IPI00008485.1	ACO1	Cytoplasmic aconitate hydratase	0.711	0.506
IPI:IPI00411706.1	ESD	S-formylglutathione hydrolase	0.479	0.501
IPI:IPI00017704.3	COTL1	Coactosin-like protein	0.466	0.501
IPI:IPI00291175.7	VCL	Isoform 1 of Vinculin	0.457	0.501
IPI:IPI00025796.3	NDUFS3	NADH dehydrogenase [ubiquinone] iron-sulfur protein 3, mitochondrial	0.540	0.497
IPI:IPI00657774.1	STX1B	Syntaxin 1B alternative isoform deltaTM	0.631	0.492
IPI:IPI00220271.3	AKR1A1	Alcohol dehydrogenase [NADP+]	0.555	0.492
IPI:IPI00006579.1	COX4I1	Cytochrome c oxidase subunit 4 isoform 1, mitochondrial	0.511	0.488
IPI:IPI00021812.2	AHNAK	Neuroblast differentiation-associated protein AHNAK	0.565	0.483
IPI:IPI00418169.3	ANXA2	Isoform 2 of Annexin A2	0.413	0.453
IPI:IPI00016077.1	GBAS	Protein NipSnap homolog 2	0.550	0.441
IPI:IPI00645031.1	CRYL1	Isoform 2 of Lambda-crystallin homolog	0.483	0.441
IPI:IPI00301180.4	SLC12A5	Isoform 2 of Solute carrier family 12 member 5	0.479	0.441
IPI:IPI00027497.5	GPI	Glucose-6-phosphate isomerase	0.705	0.433
IPI:IPI00872379.1	ANXA5	36 kDa protein	0.373	0.421
IPI:IPI00010130.3	GLUL	Glutamine synthetase	0.649	0.417
IPI:IPI00514285.2	PTGDS	Prostaglandin D2 synthase 21kDa	0.497	0.413
IPI:IPI00946099.1	SRI	Putative uncharacterized protein SRI	0.328	0.409
IPI:IPI00216138.6	TAGLN	Transgelin	0.302	0.409
IPI:IPI00013043.1	TPPP	Tubulin polymerization-promoting protein	0.685	0.394
IPI:IPI00219067.4	GSTM2	Glutathione S-transferase Mu 2	0.497	0.391
IPI:IPI00302592.2	FLNA	Isoform 2 of Filamin-A	0.350	0.391
IPI:IPI00005038.1	HRSP12	Ribonuclease UK114	0.406	0.387
IPI:IPI00514424.4	PPT1	Palmitoyl-protein thioesterase 1	0.394	0.377
IPI:IPI00641737.1	HP	Haptoglobin	0.429	0.356
IPI:IPI00303568.3	PTGES2	Prostaglandin E synthase 2	0.429	0.356
IPI:IPI00013698.3	ASAH1	N-acylsphingosine amidohydrolase (Acid ceramidase) 1, isoform CRA_c	0.433	0.353
IPI:IPI00604710.2	SLC3A2	Isoform 1 of 4F2 cell-surface antigen heavy chain	0.488	0.347
IPI:IPI00021828.1	CSTB	Cystatin-B	0.492	0.328
IPI:IPI00002280.1	PCSK1N	ProSAAS	0.313	0.328
IPI:IPI00413674.1	PHYHD1	Isoform 1 of Phytanoyl-CoA dioxygenase domain-containing protein 1	0.511	0.302
IPI:IPI00515081.4	IGSF1	Isoform 2 of Immunoglobulin superfamily member 1	0.366	0.296
IPI:IPI00423460.3	IGHA1	Putative uncharacterized protein DKFZp686G21220 (Fragment)	0.233	0.273
IPI:IPI00218487.3	GJA1	Gap junction alpha-1 protein	0.283	0.268
IPI:IPI00022143.3	ESYT1	Isoform 1 of Extended synaptotagmin-1	0.384	0.265
IPI:IPI00156689.3	VAT1	Synaptic vesicle membrane protein VAT-1 homolog	0.360	0.238
IPI:IPI00011200.5	PHGDH	D-3-phosphoglycerate dehydrogenase	0.261	0.217
IPI:IPI00027442.4	AARS	Alanyl-tRNA synthetase, cytoplasmic	0.437	0.209
IPI:IPI00010800.2	NES	Nestin	0.077	0.099
IPI:IPI00001734.3	PSAT1	Phosphoserine aminotransferase	0.067	0.086
IPI:IPI00220301.5	PRDX6	Peroxiredoxin-6	0.078	0.082

**Fig 1:**
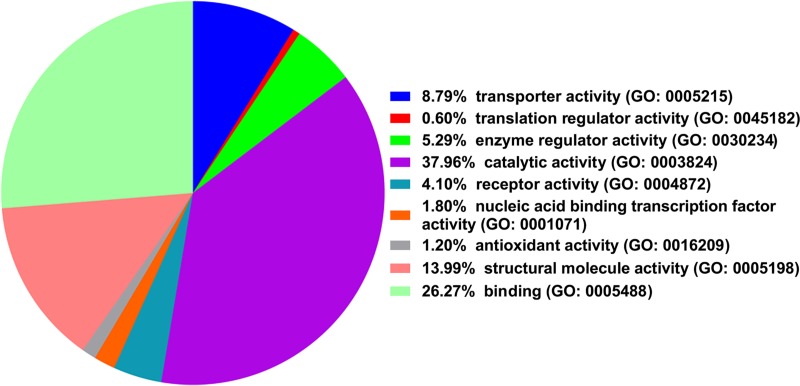
Molecule functional categories of 153 differentially exprssed proteins using the PANTHER Classification System.

Using PANTHER classification system, the 153 proteins were divided into 9 functional categories including catalytic activity (38.0%), binding (26.3%), molecule-structuring activity (14.0%), transporter activity (8.8%), and enzyme regulation activity (5.3%) (Fig 1). 8 differentially expressed proteins, including FSCN1, CRMP1, NDRG1, DPYSL5, MAP4, FABP3, PRDX6 and PSAP were selected for further validation. The gene oncology terms of these 8 proteins were shown in S1 Table.

### qPCR in children having CCD with epilepsy

**Fig 2:**
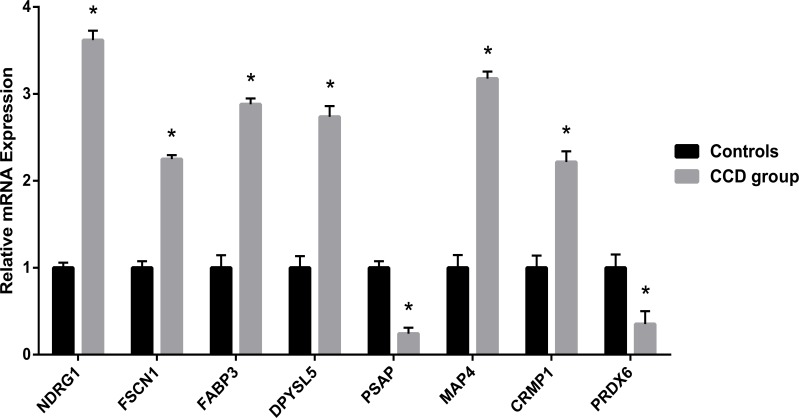
Relative mRNA expression levels of NDRG1, FSCN1, FABP3, DPYSL5, PSAP, MAP4, CRMP1, PRDX6. *p<0.05 compared to controls.

The levels of mRNA expression for fascin actin-bundling protein 1 (FSCN1), collapsin response mediator protein 1 (CRMP1), N-myc downstream regulated 1 (NDRG1), dihydropyrimidinase-related protein 5 (DPYSL5), peroxiredoxin 6 (PRDX6), prosaposin (PSAP), microtubule associated protein 4 (MAP4), and fatty acid binding protein 3 (FABP3) are presented in Fig 2. The expression of FSCN1, CRMP1, NDRG1, DPYSL5, MAP4, and FABP3 were found to be up-regulated in the CCD patients (Relative mRNA expression: CRMP1, 2.21±0.12; NDRG1, 3.61±0.11; DPYSL5, 2.73±0.12; MAP4, 3.17±0.08; FAPB3, 2.88±0.06. p<0.05 for each mRNA expression compared to controls), and the expression of PRDX6 and PSAP were down-regulated (Relative mRNA expression: PRDX6, 0.35±0.14; PSAP, 0.24±0.06. p<0.05 for both mRNA expression compared to controls) compared to controls.

### Immunoblotting

**Fig 3:**
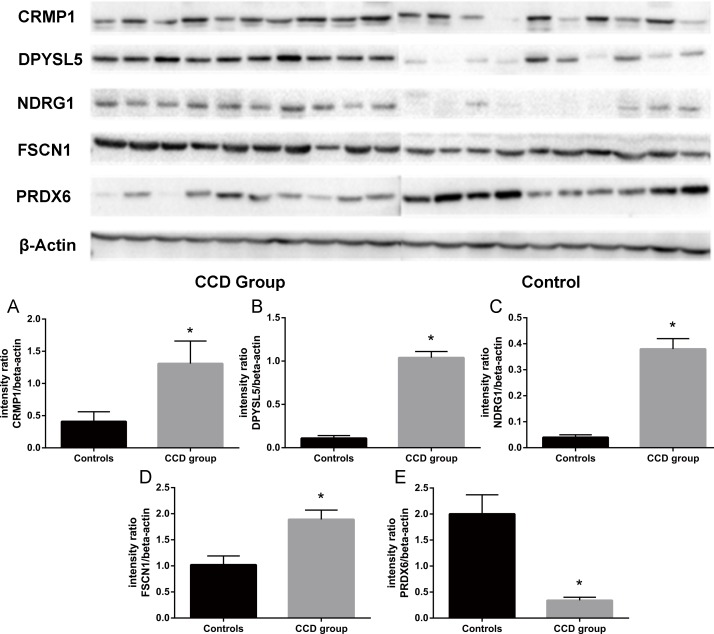
Immunoblotting for CRMP1, DPYSL5, NDRG1, FSCN1 and PRDX6. Quantification of protein levels showed increased expression of CRMP1 (A), DPYSL5 (B), NDRG1 (C) and FSCN1 (D) and decreased expression of PRDX6 (E) in brain tissue of childhood cortical dysplasia patients compared to controls. *p<0.05 compared to controls.

CRMP1, DPYSL5, FSCN1, NDRG1, PRDX6 were further validated with immunoblotting. In CCD patients, the protein levels of CRMP1 (CCD group: 1.31±0.35; Controls: 0.41±0.15. p<0.05), DPYSL5 (CCD group: 1.04±0.07; Controls: 0.11±0.03. p<0.05), FSCN1 (CCD group: 1.89±0.18; Controls: 1.02±0.17. p<0.05) and NDRG1 (CCD group: 0.38±0.04; Controls: 0.04±0.01. p<0.05) were increased, while the protein level of PRDX6 (CCD group: 0.34±0.06; Controls: 2.00±0.37. p<0.05) was decreased compared to controls (Fig 3).

### Immunohistochemistry

**Fig 4:**
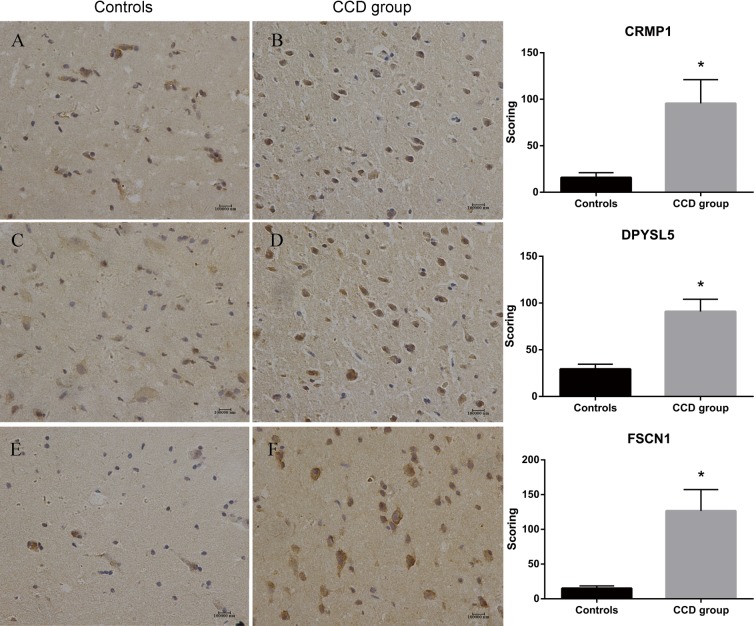
Immunohistochemistry of CRMP1, DPYSL5 and FSCN1. IHC score of CRMP1 (A & B), DPYSL5 (C & D) and FSCN1 (E & F) were significantly increased in brain tissues of childhood cortical dysplasia patients with epilepsy compared to controls. *p<0.05 compared to controls.

The expression of DPYSL5, CRMP1 and FSCN1 were further measured by IHC. There were increased immunoreactivity of CRMP1 (Scores: CCD group: 95.50±25.52; Controls: 15.76±5.28. p<0.05) (Fig 4), DPYSL5 (Scores: CCD group: 90.93±13.15; Controls: 29.33±5.21. p<0.05) (Fig 4), and FSCN1 (Scores: CCD group: 126.53±30.70; Controls: 15.06±3.45. p<0.05) (Fig 4) in the CDD group compared to controls.

## Discussion

In our study, 153 proteins were identified differentially expressed in brain tissues of CCD patients with epilepsy compared to controls using iTRAQ. According to the functional classification using PANTHER, the 153 differentially expressed proteins were divided into 9 categories, which were involved in activities of various biological process, including catalytic activity (38.0%), binding (26.3%), molecule-structuring activity (14.0%), transporter activity (8.8%), and enzyme regulation activity (5.3%).

Among the 153 proteins, the expression of FSCN1, CRMP1, NDRG1, DPYSL5, MAP4 and FABP3 was increased in the CCD patients compared to controls, while the expression of PRDX6 and PSAP was decreased in iTRAQ analysis. And these results were validated by real-time PCR, immunoblotting and immunohistochemistry.

FSCN1 is an actin-binding protein and can affect the formation and maintenance of cytoskeleton structure [22]. FSCN1 increases in neurogenesis and can help neurites maintain their normal shape, and it is considered as a candidate gene for developmental brain disorders [23, 24]. FSCN1-related pathways mainly participate in the migration of neurons, which was known as a key mechanism of cortical dysplasia [25]. So it is possible that, in our study, the increased level of FSCN1 indicates an abnormally enhanced neurogenesis, neurite outgrowth and neuronal migration, and thus, result in CCD and epilepsy. However, the effect of increased FSCN1 on neurons needs to be further researched.

Collapsin response mediator protein 1 (CRMP1) belongs to the collapsing response mediator protein family (CRMPs) which is involved in the Sema-3A signaling pathway [26–28], CRMP1 also regulates migration, neurite outgrouwth, and dendrite orientation of neurons, its loss can retard the radial migration and neurite outgrowth of neurons and lead to abnormal orientation of basal dendrites of neurons [29–31]. Similar to FSCN1, the increased level of CRMP may also possibly indicate an abnormally enhanced neuron migration and neurite outgrowth and abnormal orientation of dendrites, which may have roles in CCD. Interestingly, in temporal lobe epilepsy (TLE) patients and animal models, decreased CRMP1 expression was reported [32]. It is possible that CRMP1 plays different roles in CCD with epilepsy and TLE. The specific role of CRMP1 in CCD with epilepsy needs further evaluation.

NDRG1 is upregulated during cell differentiation, and its cellular distribution and molecular assembly changes with postnatal development, which is correlated with the maturation of brain [33]. NDRG1 exists in oligodendrocytes in cerebrum and decreases significantly at the end stage of myelin degradation [34, 35], and its mutation is found related to subcortical white matter abnormalities and severe demyelinating neuropathy [36]. Interestingly, in patients with cortical dysplasia, the change of oligodendrocytes and oligodendrocyte precursor cells is conflicting in previous reports [37, 38], and some patients with malformation of cortical development have reactive oligodendroglial hyperplasia [37]. These suggest complicated roles of oligodendrocytes and myelin sheath in cortical dysplasia. In our study, we found NDRG1 abnormally increased in CCD patients, which may suggest a possible mechanism of reactive oligodendroglial hyperplasia in CCD. However, whether NDRG1-mediated oligodendroglial change participate in the pathogenensis of CCD needs to be further illustrated.

Increased expression of DPYSL5 can regulate dendritic development by mediating BDNF signaling in the central nervous system and modulate the function of CRMP2 by interacting with tubulin [39, 40], thus affect the cytoskeleton remodeling, which is important in CCD with epilepsy. It has been reproted that BDNF, a neurotrophin, plays an important role in dendritic arborzation and synaptic neurotransmission [41–43], and CRMP2, a signaling molecule of Semaphoring-3A and a repulsive guidance cue, can induce growth cone collapse and regulate neuronal polarity [28], axon elongation and multiple axon formation [44, 45]. These suggest that DPYSL5 may function in CCD with epilepsy via affecting BDNF and CRMP2.

MAP4 exists in brain and many other organs, one of its isoforms was found neural cell specific and it can inhibit the movement of the microtubules in a concentration-dependent manner and reduce microtubule-stabilizing activity [46–48]. MAP4 is also known associated with epilepsy [49]. Notably, microtubule-associated proteins were known important in regulating neuronal migration and brain development [50]. Defects of neuronal migration can lead to cortical malformation and consequently cause severe intellectual disability and refractory epilepsy [51]. Therefore, the increase of MAP4, as in our study, may inhibit the movement and activity of microtubles and thus impair neuronal migration which participate in CCD.

FABP3 is considered as a promising and sensitive marker for minor brian injury and Creutzfeldt-Jakob disease [52, 53]. FABP3 expression is very low in neonatal brains and gradually increases after birth until adulthood, its expression pattern is correlated with synaptogenesis, myelinogenesis, neurite formation and synapse maturation [54]. FABP3 regulate the incorporation of arachidonic acid into brain, and may also regulate gene expression via controlling the availability of fatty acid ligands required for PPAR and RXR activity [54]. In our study, a increased FABP3 level was found in CCD patients, possibly indicating early maturity of metabolism pattern in CCD patients, which may contribute to the formation of cortical dysplasia. Moreover, FABP3 deficiency in mice showed protective effect against experimental autoimmune encephalomyelitis [55], indicating a possible role of autoimmune inflammation in CCD.

PRDX6 is an antioxidant protein which mainly exists in glia and keeps increasing as growing, it may have important roles in alzheimer’s disease and parkinson’s disease [56, 57]. PRDX6 can clear reactive oxygen species, regulate gene expression in brain and protect against oxidative stress-induced neuronal death [58]. Whether the reduction of PRDX6 in CCD patients is causal or consequential factor of CCD remains to be further illustrated. It is possible that, reduction of PRDX6 is a result of enhanced oxidative stress, which has been reported in previous study [58]. However, it is also possible that reduction of PRDX6 may contribute to the pathogenesis of CCD, because oxidative stress has been associated with developmental brain disorders and epileptogenesis, although the specific role of oxidative stress in the pathogenesis of cortical dysplasia remains to be illustrated [59, 60].

PSAP is precursor of saposin and acts as a lysosomal protein and a potent secreted neurotrophic factor, its temporal pattern of expression in perinatal brain indicate its potential role in brain development [61]. Infants with PSAP deficiency presented multifocal myoclonus and cyanotic hypoxemia immediately after birth, grand-mal epilepsy in the following days, and cortical and white matter morphogenetic disorders [62, 63]. This deficiency is considered to cause such manifestations via impairing the lipid storage[62, 63]. Therefore, abnormally reduction of PSAP in CCD patients may indicate a possible role of PSAP in the pathogenesis of CCD. Moreover, in kainate-induced epilepsy models, PSAP reactively increases and protects against the neurotoxicity [64]. Thus, PSAP reduction in CCD may also participate in the neuronal damage in CCD.

In conclusion, we identified 153 differentially expressed proteins in CCD patients compared to controls. Among these proteins, FSCN1, CRMP1, NDRG1, DPYSL5, MAP4, FABP3, PRDX6 and PSAP were further validated. These proteins have not been related to CCD before. Mechanisms including neuronal migration, neurite growth, cytoskeleton remodeling, inflammation, oligodendroglia hyperplasia, metabolic pattern and lipid storage may be involved in CCD pathogenesis and/or pathophysiology via these proteins, providing potential targets and directions for future researches on cortical dysplasia. Our study also indicate a complicated pathogenetic background of CCD, as these differentially expressed proteins have various cellular distribution and function. Moreover, further study is needed to illustrate the specific effects of these differentially expressed proteins on CCD with epilepsy, considering the limited sample size due to the critical criteria of surgery in CCD patients, especially in children.

## Supporting information

S1 Table Gene oncology terms of the FSCN1, CRMP1, NDRG1, DPYSL5, MAP4, FABP3, PRDX6 and PSAP. (DOC)

S1 Datasheet The raw data of iTraq-MS. (XLSX)

S1 Text STROBE checklist. (DOCX)
