# Supplementary material for: Differentially expressed proteins underlying childhood cortical dysplasia with epilepsy identified by iTRAQ proteomic profiling
Source: PLoS One. 2017 Feb 21;12(2):e0172214. doi: 10.1371/journal.pone.0172214 (PMC5319751; doi:10.1371/journal.pone.0172214)
Supplement: S1 Table — (DOC) [file pone.0172214.s001.doc]

**S1 Table.** Gene oncology terms of the FSCN1, CRMP1, NDRG1, DPYSL5, MAP4, FABP3, PRDX6 and PSAP.

| Gene name | Gene oncology terms | |
| --- | --- | --- |
|  | Function | Process |
| FSCN1 | actin binding; actin filament binding; cadherin binding involved in cell-cell adhesion; drug binding; poly(A) RNA binding; protein binding; protein binding, bridging | actin cytoskeleton organization; actin filament bundle assembly; anatomical structure morphogenesis; cell migration; cell motility; cell proliferation; cell-cell adhesion; cell-cell junction assembly; establishment of apical/basal cell polarity; microspike assembly; positive regulation of extracellular matrix disassembly; positive regulation of filopodium assembly; positive regulation of filopodium assembly; positive regulation of lamellipodium assembly; positive regulation of podosome assembly; regulation of actin cytoskeleton organization; regulation of microvillus assembly |
| CRMP1 | filamin binding; hydrolase activity, acting on carbon-nitrogen (but not peptide) bonds; protein binding | axon guidance; microtubule cytoskeleton organization; negative regulation of actin filament binding; negative regulation of neuron projection development; nervous system development; nucleobase-containing compound metabolic process |
| NDRG1 | Rab GTPase binding; cadherin binding; cadherin binding involved in cell-cell adhesion; gamma-tubulin binding; microtubule binding; protein binding | DNA damage response; signal transduction by p53 class mediator; cell-cell adhesion; cellular response to hypoxia; mast cell activation; negative regulation of cell proliferation; peripheral nervous system myelin maintenance; positive regulation of spindle checkpoint; regulation of apoptotic process; response to metal ion |
| DPYSL5 | hydrolase activity, acting on carbon-nitrogen (but not peptide) bonds; microtubule binding; protein binding | axon guidance; nervous system development; signal transduction |
| MAP4 | microtubule binding; poly(A) RNA binding; protein binding; structural molecule activity | cell division; establishment of spindle orientation; establishment of spindle orientation; microtubule sliding; mitotic spindle organization; negative regulation of non-motile cilium assembly; neuron projection development |
| FABP3 | cytoskeletal protein binding; icosatetraenoic acid binding; long-chain fatty acid binding; long-chain fatty acid transporter activity; oleic acid binding; protein binding | cholesterol homeostasis; fatty acid metabolic process; long-chain fatty acid import; long-chain fatty acid transport; negative regulation of cell proliferation; phospholipid homeostasis; positive regulation of phospholipid biosynthetic process; regulation of fatty acid oxidation; response to drug; response to fatty acid; response to insulin; triglyceride catabolic process |
| PRDX6 | cadherin binding involved in cell-cell adhesion; glutathione peroxidase activity; hydrolase activity; peroxiredoxin activity; protein binding; ubiquitin protein ligase binding | cell redox homeostasis; cell-cell adhesion; hydrogen peroxide catabolic process; lipid catabolic process; oxidation-reduction process; response to reactive oxygen species |
| PSAP | G-protein coupled receptor binding; enzyme activator activity; lipid binding; protein binding | adenylate cyclase-inhibiting G-protein coupled receptor signaling pathway; cellular response to organic substance; epithelial cell differentiation involved in prostate gland development ; glycosphingolipid metabolic process; lipid transport; negative regulation of hydrogen peroxide-induced cell death; platelet degranulation; positive regulation of MAPK cascade; positive regulation of catalytic activity; prostate gland growth; regulation of autophagy; regulation of lipid metabolic process |
